# Supplementary material for: CMV-Specific Immune Response—New Patients, New Insight: Central Role of Specific IgG during Infancy and Long-Lasting Immune Deficiency after Allogenic Stem Cell Transplantation
Source: Int J Mol Sci. 2019 Jan 11;20(2):271. doi: 10.3390/ijms20020271 (PMC6358762; doi:10.3390/ijms20020271)
Supplement: Supplementary file 1 [file ijms-20-00271-s001.pdf]

Supplementary Material

Table S1. List of CMV T-cell epitopes used in the quantiferon technique.

Epitope Sequence	HLA Restriction	Epitope Code	Antigen
IMREFNSYK	A3	IMR	gB
VLEETSVML	A2	VLE	IE-1
QIKVRVDMV	B8	QIK	IE-1
AYAQKIFKIL	A23&A24	AYA	IE-1
FEQPTETPP	B41	FEQ	IE-2
VTEHDTLLY	A1	VTE	pp50
TPRVTGGGAM	B7	TPR	**pp65**
QYDPVAALF	A24	QYD	**pp65**
QMWQARLTV	B52	QMW	**pp65**
QEFFWDANDI	B44	QEF	**pp65**
QAIRETVEL	B57&B58	QAI	**pp65**
PFTSQYRIQGKL	A24	FTS	**pp65**
NLVPMVATV	A2	NLV	**pp65**
KMQVIGDQY	B40/60	KMQ	**pp65**
GPISGHVLK	A11	GPI	**pp65**
FPTKDVAL	B35	FPT	**pp65**
DIYRIFAEL	A26	DIY	**pp65**
DALPGPCI	B51	DAL	**pp65**
CPSQEPMSIYVY	B35	CPS	**pp65**
CEDVPSGKL	B40/60	CED	**pp65**
ARVYEIKCR	B27	ARV	**pp65**

Current immunoassays use a broad spectrum of CMV-antigen types (synthetic peptides, viral lysates, recombinant antigens). However, such materials have, to date, not been issued as an official WHO standard. Of note, in quantiferon analysis, most of the epitopic peptides are derived from **pp65** (i.e. 15 to highlight presented in the bold font) , but only one was epitope from immunodominant surface glycoprotein B (gB), sometimes used in the pentamer technique. Most notably, HLA specificities are not uniformly distributed e.g., only one HLA-A3-restriced epitope (blue), contrary to 3 -HLA-A24-restricted (red). It may be a source of inter-individual variation. Under the assumption of the quantiferon technique, such HLA-restricted CMV epitopes acts via TCR to activate T cell immune response and cytokine release. Limitations of CMV-interferon gamma release assay (IGRA) and a high rate of intermediate results [11,13,14,32] were described, but IL-2 as a T cell specific cytokine yielded inferior results and was negligible as a surrogate marker [32]. The IFNγ release was significantly higher (1324 or 5871 pg/mL) than IL-2 (44 or 883 pg/mL) under the influence of recombinant or synthetic pp65 peptides [32].
